# Anatomical Mapping of the Masseter for Safe Botulinum Toxin Injection: A Cadaveric Study

**DOI:** 10.1155/bmri/5591406

**Published:** 2026-04-03

**Authors:** Yuqi Zhao, Jinran Chen, Jinrui Jiang, Zechuan Wang, Houen Zhou, Wendi Xu, Bo Xue, Liujun Yong

**Affiliations:** ^1^ School of Clinical Medicine, Chengdu Medical College, Chengdu, Sichuan, China, cmc.edu.cn; ^2^ Department of Human Anatomy, Chengdu Medical College, Chengdu, Sichuan, China, cmc.edu.cn

**Keywords:** anatomical structure, botulinum toxin, masseter, triangular zone

## Abstract

**Background:**

Botulinum toxin (BTX) is commonly used in masseter injections to enhance facial contour. However, the injection may harm the surrounding structure due to insufficient research on the anatomical structure of the masseter region.

**Objectives:**

This study is aimed at providing anatomical evidence for facial injections in clinical practice by dissecting the superficial masseter structure and analyzing it by classifying the masseteric nerve and structure.

**Methods:**

Select 24 adult gross specimens with intact facial characteristics and dissect the masseter regions to expose the surface structure of the masseter muscle. After that, draw a heatmap according to the frequency of occurrence of the above structures. Then, dissect and document the structure relationship of the masseteric nerves and each layer of the masseter.

**Results:**

The masseter region has a safe triangular zone (△LMF). The course probability of the facial nerve, parotid gland, parotid duct, facial artery, and facial vein is the lowest in this region. The masseteric structure is classified into three types: overlapped type, juxtaposed type, and parallel type, of which the juxtaposed type is the most common. In the safe triangular zone, the small branches of the masseteric nerve are mainly distributed in the connection range of line LM.

**Conclusion:**

The three‐point injection approach is presented after a comprehensive analysis incorporating the masseter injection’s safe triangular zone and the masseter’s internal structure. It provides a more accurate treatment strategy for the masseter microplastic injection.

## 1. Introduction

Among Asians, a smooth oval face is considered an attractive face shape [1]. From a morphological perspective, the masseter surrounds the mandibular angle. As the masseter is located superficially and has a relatively large volume, it is an essential anatomical component that supports the soft tissues of the mandibular angle. The types and anatomical characteristics of the masseter are critically involved in determining the morphology of the facial skeleton and significantly influence facial esthetics [2, 3]. Masseter muscle prominence (MMP) typically leads to the widening of the inferior facial part, thus influencing facial appearance [4]. Botulinum toxin (BTX) can inhibit the release of acetylcholine from the presynaptic membrane, hence blocking the signal transduction of the neuromuscular junction, which will paralyze the muscle of the injected area [5]. BTX has seven serotypes, of which botulinum toxin type A (BTX‐A) is extensively applied in clinical treatment [6] and is the major type of BTX used to treat MMP. Although using BTX‐A to treat MMP may have some aftereffects. Notably, recent studies have highlighted adverse effects on bone tissue, reporting that injection‐induced muscle atrophy and subsequent mechanical unloading can lead to bony changes in the mandibular condyle and angle [7, 8].

The location of the facial nerve, parotid gland, parotid duct, facial artery, and facial vein is crucial for facial surgical procedures. The trajectories of the parotid gland, parotid duct, facial artery, and facial vein exhibited a consistent anatomical pattern. Although the course of the facial nerve branches is variable, it has a relatively constant relationship with the soft tissue plane [9, 10]. Due to the individual differences in the facial anatomical structure, it will lead to various injection sites when applying BTX‐A to inject the masseter to treat MMP in clinical practice [11, 12]. In clinical practice, relying solely on experience to localize injection sites can lead to relatively large errors, thus causing adverse responses [13, 14]. Therefore, the injection sites should be further improved to ensure the therapeutic efficacy of BTX‐A while minimizing potential complications.

Based on the above background, this study identifies the optimal sites for masseter injection. It supports the microplastic injection of the masseter with anatomical evidence by meticulously dissecting the parotid–masseteric region of the gross specimen, analyzing the course pattern of its superficial structure, and combining the relationship between the masseter structure and the masseteric nerve.

## 2. Materials and Methods

This study selects 24 adult cadaver specimens of Han nationality with 48 sides (12 male cases and 12 female cases, with an average age of 67 years) from the southwestern region. These specimens range in age from 35 to 87 years old and have intact facial characteristics fixed with 4% formaldehyde. These gross specimens are supplied voluntarily with the informed consent of the donors and their families.

Expose the parotid gland, parotid duct, zygomatic branch of the facial nerve, buccal branch, marginal mandibular branch, facial artery, and facial vein successively in the masseter region. Then, isolate the above structures to fully expose the masseter. And dissect away the superficial masseter to expose the main trunk of the masseteric nerve and dissect downward to trace the branches of different levels. The middle and deep masseter should also be dissected, and the structure relationship of the three layers of the masseter should be observed and documented. At the inferior one‐third part of the masseter, the distance should be measured, respectively, from the posterior edge of the middle masseter to the anterior boundary of the masseter and from the anterior edge of the deep masseter to the posterior boundary of the masseter. All anatomical steps are recorded by a camera and uploaded to Microsoft Word 2021. A rectangular coordinate system in the sagittal plane is established by using the intersection of the posterior edge of the mandibular ramus and the inferior edge of the mandible as the original point, the vertical line from the original point to the inferior edge of the zygomatic arch as the positive *y*‐axis direction, and the forward direction as the positive *x*‐axis. In the coordinate system, the courses of the facial nerve, parotid gland, parotid duct, facial artery, and facial vein are statistically examined. Excel is utilized to arrange the data. After summarizing the data, GraphPad Prism 9.5.0.730 is used to make a heatmap.

Respectively, name the lateral canthus, the midpoint of the philtrum, the intertragic notch, the oral commissure, the inferior edge of the earlobe, and the mandibular angle in the order of letters A–F. And connect lines AF, BC, DC, and DE. Line AF, respectively, intersects lines BC, DC, and DE at points G, H, and I. Then, draw a perpendicular line from point F to line DE, with the foot at point J. The △LMF is formed by taking point L from the upper one‐third of line JF and extending it horizontally for half of the average width of the masseter to point M.

## 3. Results

### 3.1. The Applied Anatomy of the Superficial Structure of the Masseter

After horizontally passing through the temporomandibular joint, the main trunk of the zygomatic branch of the facial nerve divides into an upper branch and a lower branch. The upper branch ascends, following the inferior edge of the zygomatic arch. The lower branch descends and typically connects with the communicating branches of the buccal branch of the facial nerve. The buccal branch of the facial nerve has multiple branches, with numerous communicating branches that intersect with the parotid gland. The facial nerve marginal mandibular branch passes through near the mandibular angle and extends along the mandibular body’s inferior edge on the masseter surface. It typically has more than two branches (Figure 1). Each of the branches intersects with the facial vein. The parotid gland covers the surface of the masseter, originating from the inferior edge of the zygomatic arch and terminating above the mandibular angle. The majority of the parotid gland is located behind the AF line in the preauricular area (Figure 2). The parotid duct passes through the parotid gland above the middle part of the masseter. It can be classified into Type I and Type II based on its beginning point. According to the observation of the heatmap, the Type I parotid duct typically passes through horizontally in the region of △DHI. The Type II parotid duct extends diagonally upward (27.30 ± 6.09^°^) between lines BC and CD. No parotid gland or parotid duct passes through the masseter region, where line DI and line FI are connected. The course directions of the facial arteries and veins are approximately the same, typically extending along the anterior edge of the masseter. The facial veins are located behind the facial arteries (Figure 3). Fifty percent of facial veins pass through the anterior edge of the masseter without accompanying the facial arteries; 27.3% of facial arteries and facial veins simultaneously enter the masseteric plane at the anterior one‐fourth of the masseter near the inferior edge of the mandible; and 22.7% of facial arteries and facial veins extend directly toward the area around the ala nasi without passing through the masseter.

Figure 1(a) The relative frequency of the course of the facial nerves in the masseter region (left) and (b) the distribution heatmap (right).

**Figure:**
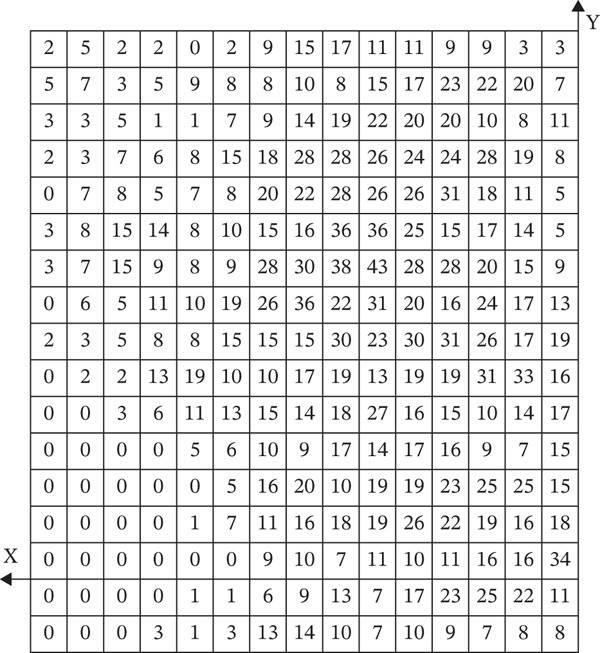
(a)

**Figure:**
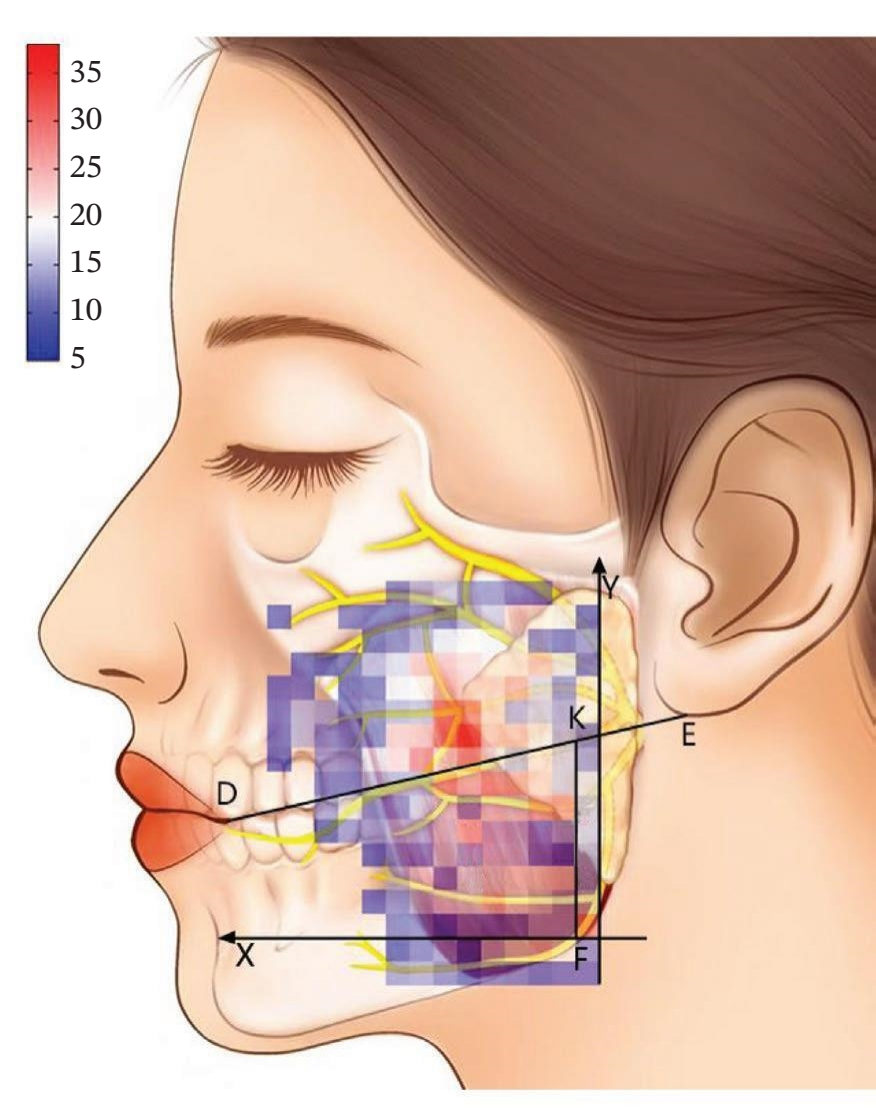
(b)

Figure 2(a) The relative frequency of the course of the parotid gland and parotid duct in the masseter region (left) and (b) the distribution heatmap (right).

**Figure:**
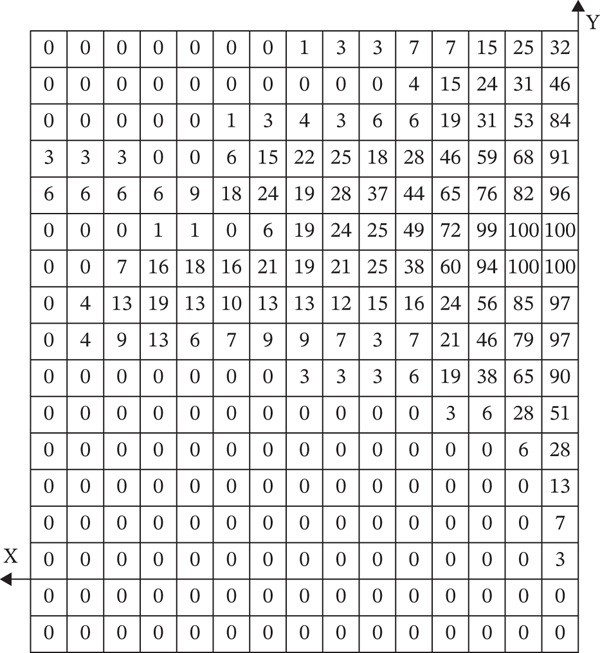
(a)

**Figure:**
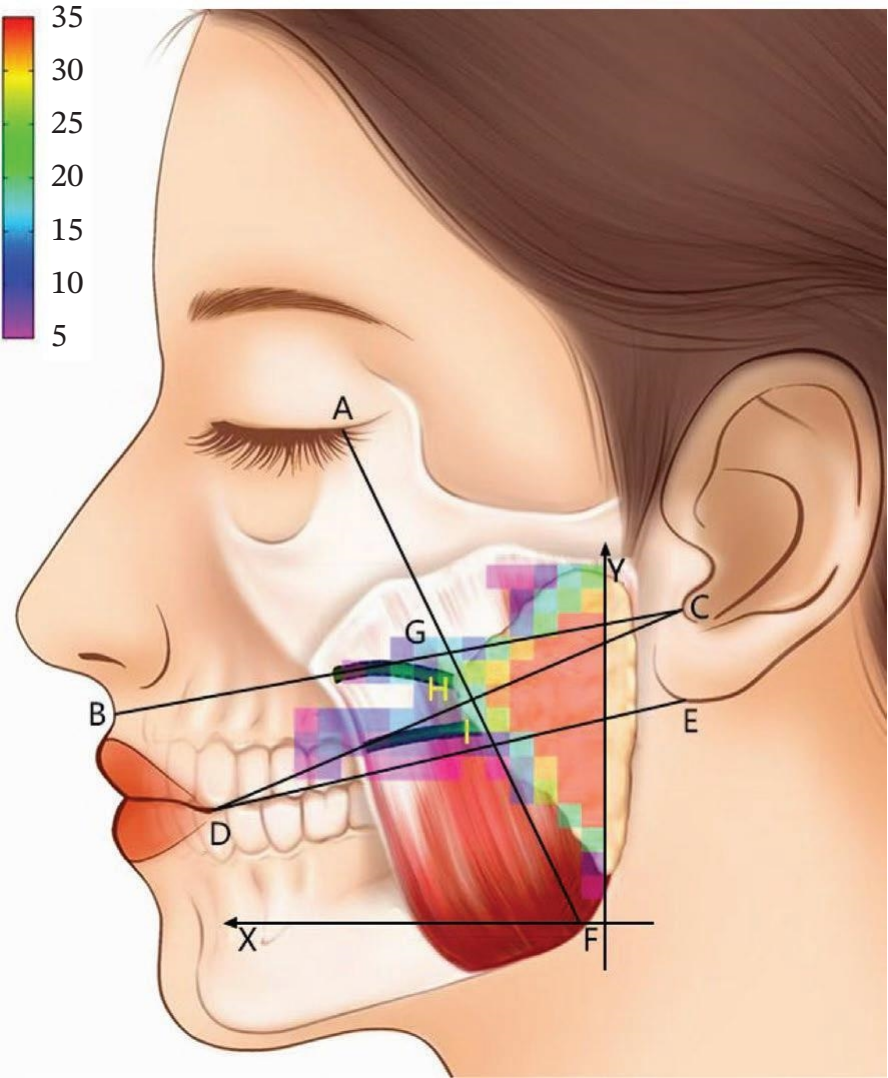
(b)

Figure 3(a) The relative frequency of the course of the facial arteries and facial veins in the masseter region (left) and (b) the distribution heatmap (right). Right figure: facial artery (red) and facial vein (blue).

**Figure:**
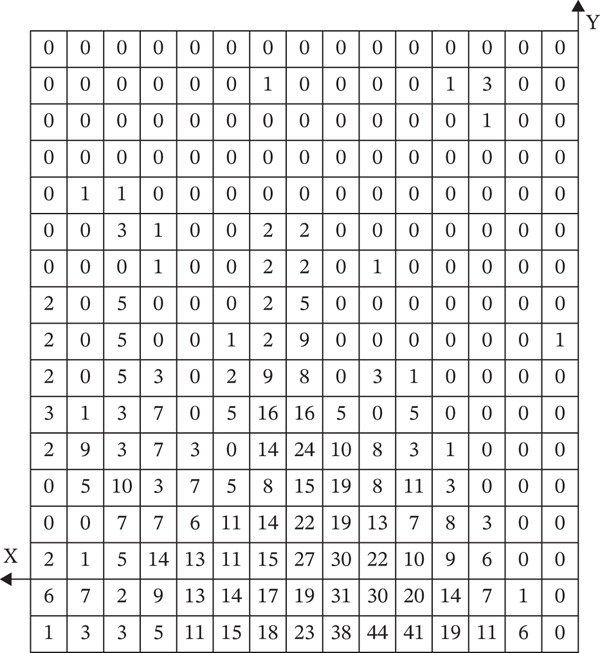
(a)

**Figure:**
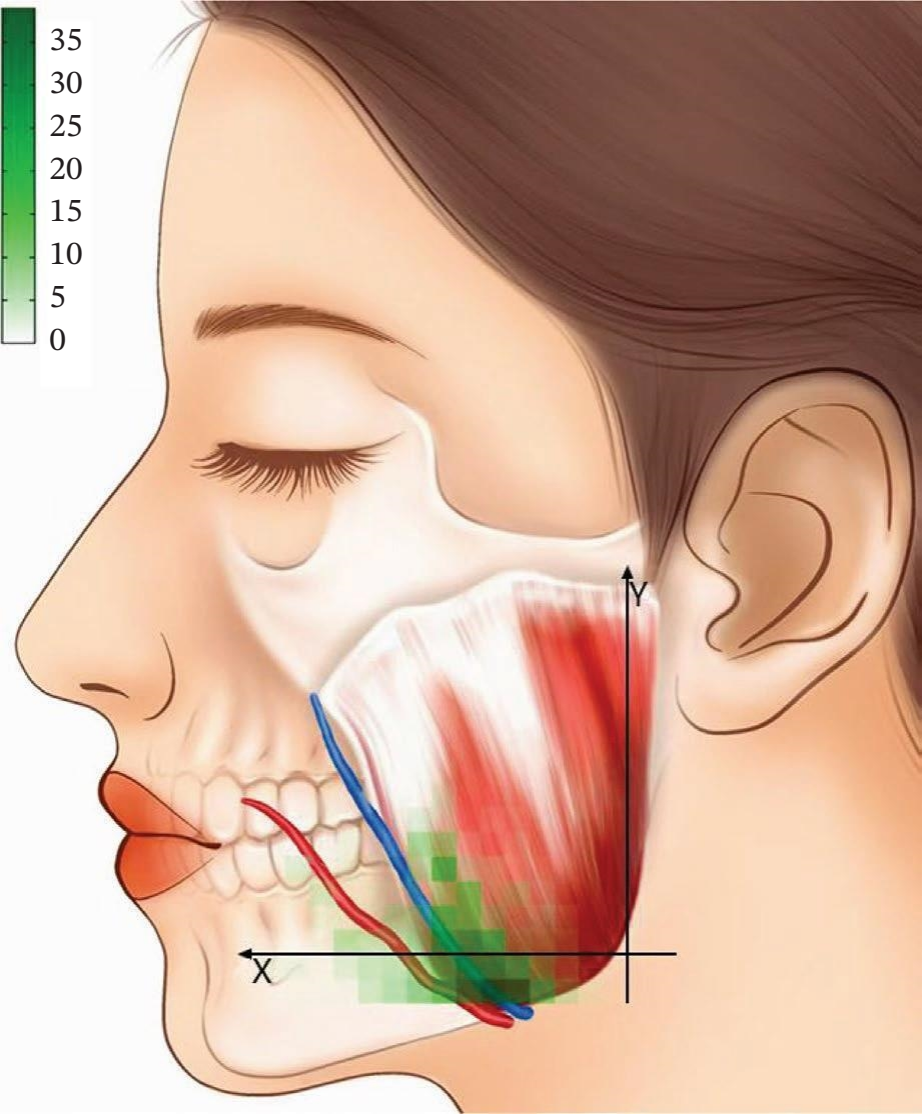
(b)

**Figure 4:**
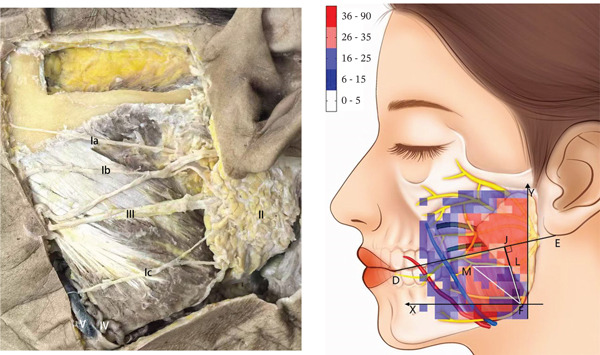
The summarized heatmap of the course of the facial nerves, parotid gland, parotid duct, and facial arteries and facial veins. The △LMF (white) is the safe triangular zone of the masseter injection.

By summarizing the course pattern of the facial nerve, parotid gland, parotid duct, facial artery, and facial vein, a course heatmap of the structures in the masseter region was drawn. In the region of △LMF, the course probability of the facial nerve, parotid gland, parotid duct, facial artery, and facial vein is relatively low, making it a safe triangular zone for masseter injection (Figure 4).

### 3.2. The Masseteric Structure and the Classifications and Applied Anatomy of the Dominant Nerve

The masseter originates from the zygomatic bone and zygomatic arch and terminates at the inferior part of the mandibular ramus, wrapping around the mandibular angle. It can be divided into three layers: superficial, middle, and deep. According to the arrangement relationship among the three layers of the masseter, it can be classified as follows: overlapping type, juxtaposed type, and parallel type. The overlapping type refers to the middle and deep layers overlapping at the junction, accounting for 41.66%; the juxtaposed type means that the middle and deep layers are located in the same plane, and the muscle bundle at the junction has a clear boundary without overlapping, accounting for 54.17%; and the parallel type means that the superficial, middle, and deep layers are clearly stratified and covered layer by layer, accounting for 4.16%. In the bottom one‐third of the masseter, the distance between the middle masseter’s posterior edge and the whole masseter’s anterior edge is 18.98 ± 5.97 mm, accounting for 35.32% of the masseter’s width. The distance from the deep masseter’s anterior edge to the whole masseter’s posterior edge is 23.53 ± 4.75 mm, which accounts for 43.68% of the masseter’s width. Then, a sketch map of the middle and deep masseter division is established after summarizing the data (Figure 5).

Figure 5(a) The masseter muscle layer diagram (left) and (b) the location relationship diagram of the surface projection and safe zone of the middle masseter and deep masseter (right). Left figure: superficial masseter (1), middle masseter (2), and deep masseter (3). Right figure: the intersection of the middle masseter and deep masseter (solid line), the posterior edge of the middle masseter (short dashed line), the anterior edge of the deep masseter (long dashed line), the overlapping area of the middle masseter and △LMF (yellow), and the overlapping area of the deep masseter and △LMF (green).

**Figure:**
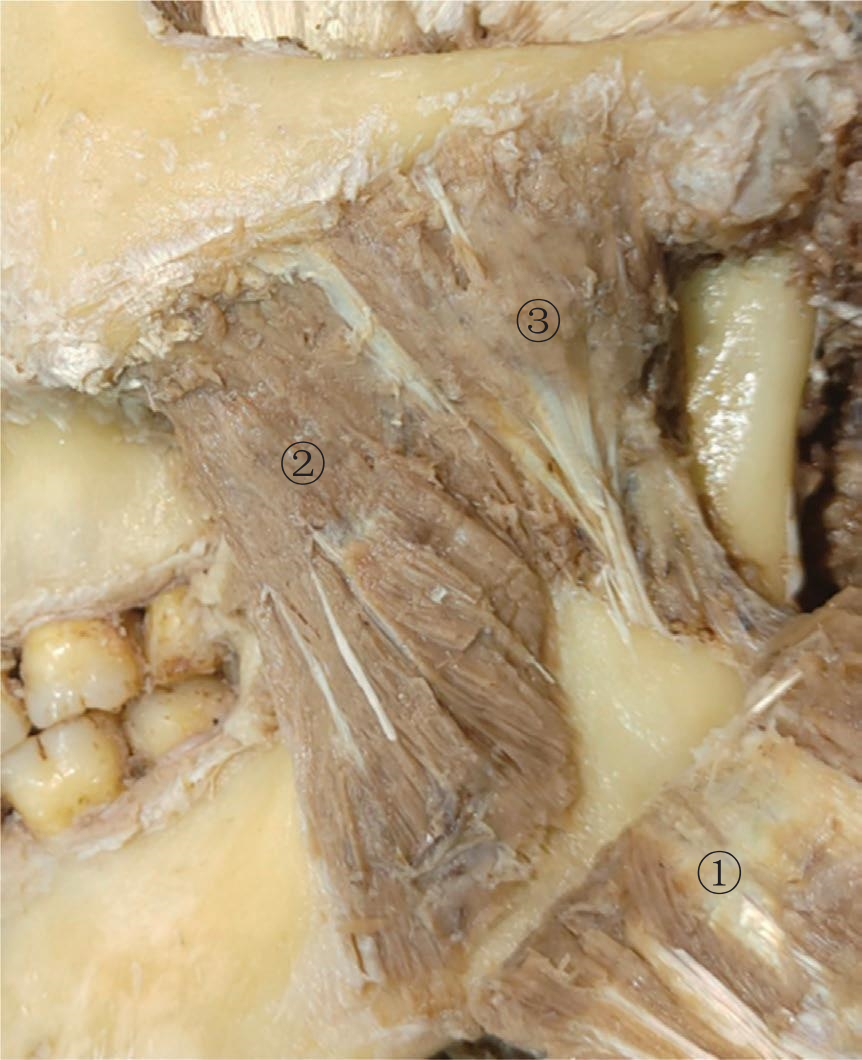
(a)

**Figure:**
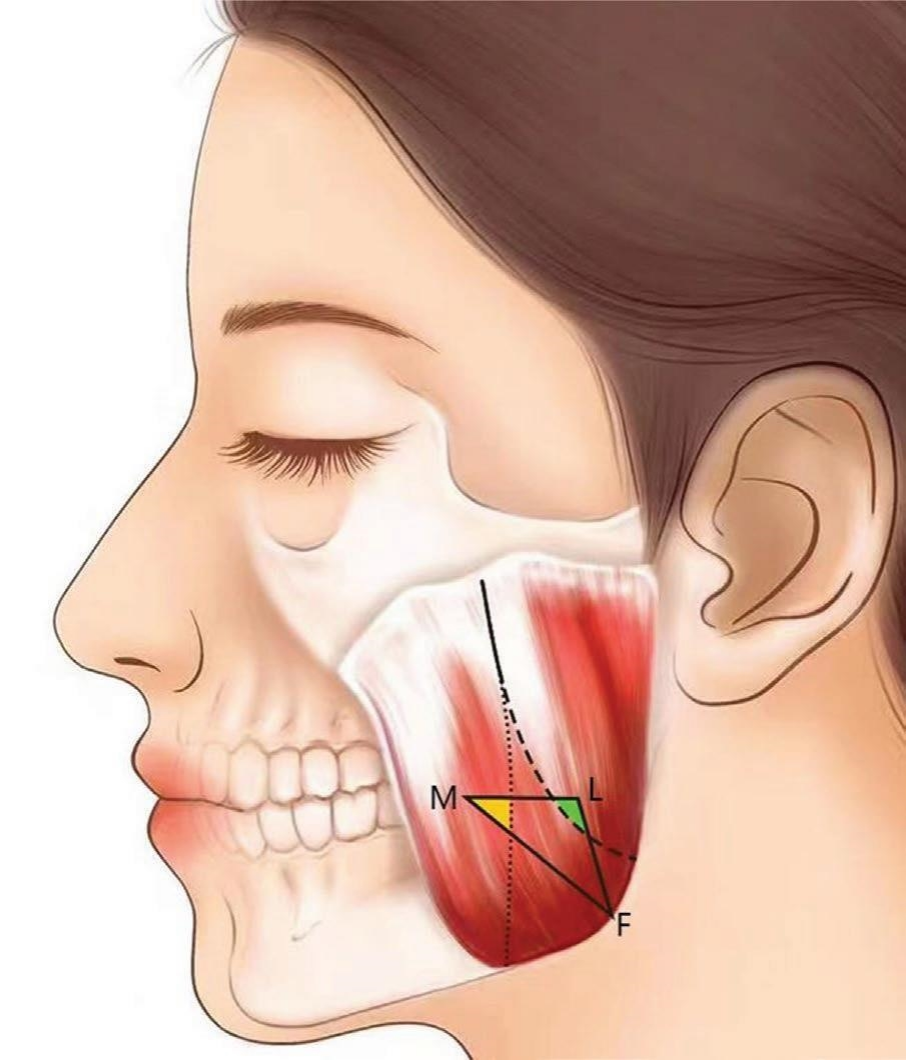
(b)

Anatomical findings show that the masseteric nerves pass through the lateral pterygoid muscle, span across the mandibular notch, and enter the masseter through the deep surface of the masseter. Then, they extend downward in the internal masseter from the posterosuperior direction of the deep layer to the medial‐inferior direction and eventually terminate at the anterior‐inferior part of the superficial masseter. The nerve trunks of the masseter mostly diverge from the middle and deep masseter intersection. Accordingly, they can be divided into single‐trunk, double‐trunk, and triple‐trunk types. In these three types, the single‐trunk type of nerves, except for the main trunk, refers to all tiny branches, accounting for 12.50%; the double‐trunk type of nerves means that the main trunk is divided into two branches, respectively, innervated to the superficial masseter and middle masseter or just to the superficial masseter, accounting for 79.16%; and the triple‐trunk type of nerves sends three branches to, respectively, innervate the masseter’s three layers, accounting for 8.33%. In the safe triangular zone of △LMF, the thickest part of the masseter is located around angle F. There are overlapping areas near angle M and angle L with the middle masseter and deep masseter, respectively, and most of the masseteric nerves transmit tiny branches into different layers of the masseter within the connection range of line LM (Figure 5).

## 4. Discussion

Moore and Wood [15] first treated MMP by injecting BTX into the most prominent area of the masseter in 1994. Since then, BTX has been routinely utilized in masseter injection to enhance patients’ appearance. However, the intricate facial anatomy leads to a high risk of injection‐related complications. Symptoms such as decreased masseter masticatory power, facial depression, paradoxical masseteric bulging (PMB), local swelling, blood stasis, and neurological injury were frequently reported as a result of improper injection [14]. Blind injections into the masseter carry inherent risks. In contrast, ultrasound guidance allows for separate visualization of the three muscle parts, ensures more precise and safer injections, and facilitates follow‐up through measurements of muscle thickness and volume [16]. Currently, research on masseter injection is primarily centered on ultrasonic localization scanning or simply dissecting the primary structure on the surface of the masseter while ignoring its interior anatomy. The purpose of this study is to describe the safe zone map of masseter injection by summarizing heatmaps and to provide anatomical data support for clinical injections by dissecting the superficial structure of the masseter and combining it with the masseteric structure and masseteric nerves.

The facial nerve, parotid gland, parotid duct, facial artery, and facial vein course over the superficial masseter. Previous studies only identified the safe zone of masseter injection by anatomical mapping. They lacked a quantitative description of the anatomical variation of the injection region, potentially leading to erroneous identification of risk areas in clinical practice. On the other hand, the heatmap application presented by Guo et al. [17] in their study of the vascular course offers a fresh perspective. As a result, this study uses a similar way to visualize the safe zone of BTX‐A injection. However, Guo et al. used a 5 × 5 mm coordinate system to localize facial arteries and veins. This strategy, which localizes through distance, will be influenced by individual anatomical variations and operators’ expertise in clinical practice. It may cause a loss of localization accuracy. So, this study equally divides the mandibular ramus into 15 pieces to establish a coordinate system, creates a heatmap, and localizes structures by utilizing landmarks to improve the operability of clinical practice. The advantage of using a heatmap to research the facial nerve, parotid gland, parotid duct, facial artery, and facial vein is that it visually displays the probability distribution in the anatomy of the masseter injection region and the common locations and variation ranges of different anatomical structures. This technology allows doctors to more correctly identify and avoid high‐risk regions, which reduces the occurrence of injection‐related complications.

Presently, clinical practice frequently views the sections below the line linking the oral commissure to the earlobe, as well as the anterior and posterior edges of the masseter and the inferior edge of the mandibular body as the boundary of the traditional safe zone of masseter injection [18]. However, according to the findings of this study, because the traditional safe zone covers the course of the buccal branch of the facial nerve, the marginal mandibular branch, the parotid gland, and the facial vein, medications and acute injuries from injections would cause harm to these structures. According to this study, the parotid gland is localized behind the line connecting the lateral canthus to the mandibular angle, and the parotid duct extends in the region of two lines: from the oral commissure to the inferior edge of the earlobe and from the midpoint of the philtrum to the intertragic notch. This conclusion is broadly in line with the findings of Uzmansel et al. [19] and Stringer et al. [20]. Therefore, the surgery in the parts anterior to the line linking the lateral canthus to the mandibular angle, as well as under the line from the oral commissure to the inferior edge of the earlobe, does not harm the parotid gland and parotid duct normally. However, in the traditional safe zone, the parotid gland below the line linking the oral commissure to the earlobe’s lower edge is easily neglected. Some academics have also noted that if the injection site is exceedingly close to the posterior edge of the masseter, it will result in parotitis [21]. Accordingly, imprecise localization below this line during injection is likely to injure the parotid gland. The anterior edge of the masseter is primarily the course area of the facial artery and facial vein. This study also finds that only 22.7% of facial arteries and facial veins do not pass through the anterior edge of the masseter, and most are attached to the anterior edge of the masseter. The facial veins extend behind the facial arteries and have an overlap of 0.2–1.0 cm with the most anterior attachment point of the masseter [9]. So, when doing the masseter injection, the facial vein is more likely to be injured than the facial artery. Therefore, injections on the anterior edge of the masseter should be avoided. In subsequent research, Duan et al. [22] proposed that multiple‐spot injections be controlled at a distance of no less than 1 cm from the boundary of the traditional safe zone, reducing the likelihood of injuring the facial nerves, parotid gland, and facial veins to a certain extent. However, they did not specify the number of injection sites, while Peng and Peng [23] advocated injecting three to four points in this range with an interval of no less than 1 cm between each point. Although their suggestions had been put forward, the range and sites for injecting BTX‐A into the masseter have not yet been standardized.

Currently, one‐to‐six‐point masseter injection approaches are all used in clinical practice. Rauso et al. [24] advocated that the fewest injection sites should be utilized as far as possible to avoid patient discomfort and limit aftereffects. However, the three‐point injection remains the most often used approach in clinical practice to guarantee the therapeutic effect. However, there are differences in the injection sites for the three‐point injection approach. Li et al. [25] advocated localizing at approximately 2 cm above the marginal mandibular and in the region of the anterior and posterior edges of the masseter to inject at three points. The first point should be done at the most prominent part of the masseter and inject 50% of the unilateral dose; the other two points should form an isosceles triangle with the first point and, respectively, inject 25% of the drug dose. However, according to this study, injecting in this range is likely to cause harm to the facial vein and the marginal mandibular branch of the facial nerve. Ryoo et al. [26] advised that the injection should be done at the thickest part of the masseter within the safe zone. The succeeding two injections should be, respectively, done above and below the reference injection site with a distance of 1–1.5 cm to guarantee drug diffusion. However, due to the relatively large individual variations in masseter volume and the complex courses of the facial nerve, parotid gland, and facial artery and vein, localizing the injection site by distance or experience may result in relatively large errors. After dividing the masseter into eight areas, Hu et al. [27] suggested that injecting in Region VI can prevent damage to the surface structure of the masseter. This result nearly matches the safe triangular zone of △LMF of the masseter injection in this study, which was achieved by connecting the auxiliary line to the landmark. However, the research did not take into account the masseteric structure. Injecting in this area may only paralyze the superficial and middle masseter and may not affect the deep masseter.

Most current research determines the masseter’s safe injection sites based on the anatomical structure of the masseter surface or clinical experience. However, the complicated anatomical structure of the masseter remains one of the sources of complications such as PMB produced by uneven diffusion of BTX‐A in the internal masseter after injection [28]. Thus, the internal anatomical structure of the masseter during the injection procedure should not be overlooked. Clinical practice typically chooses the most prominent part of the masseter as one of the injection sites [29]. However, as the masseter contracts, several types of muscle prominence exist. Some patients’ masseters have two or three prominent parts, and the localizations of these parts are not fixed [30]. As a result, there are individual differences in this injection site. The masseter comprises three layers: superficial, middle, and deep, each with its own fiber composition [31], morphological characteristics, and course direction [32]. According to earlier research, the inferior one‐third part of the masseter is the thickest, composed primarily of the masseter belly of the superficial masseter. In clinical practice, the highest bulge of the masseter during contraction is typically located near the mandibular angle. As a result, if the most prominent part of the masseter is located outside the safe triangular zone of △LMF during injection, a point within angle F can be chosen for an oblique needle insertion. After the insertion, injecting the uplift area can ensure effective treatment while avoiding injury to the critical structure. This experiment presents the masseteric structure and classifications based on anatomical observations and statistics of the arrangement relationship of the three layers of the masseter. Among the three proportions of the masseteric structure, the juxtaposed type of the masseter is the most prevalent, followed by the overlapping type, and the parallel type is the least prevalent. After combining the safe zone with the masseteric structure, it is discovered that the middle masseter has an overlapping area with angle M, and the deep masseter has an overlapping area with angle L. As a result, injecting into the overlapping areas of angle L and angle M can, respectively, penetrate the superficial masseter and middle masseter and the superficial masseter and deep masseter. In addition, because the area around line LM is typically the boundary of the middle masseter and deep masseter and there are several tiny branches sent by the masseteric nerves, injecting here can theoretically allow for a lower medicine dose and maximally block the masseteric nerves. And given that the aforementioned two sites are located in the middle part of the masseter, near the temporal muscle, the drug dose should be reduced during injection to lessen the diffusion risk. As a result, this study, which combines the masseteric structure with the masseteric nerve based on the safe zone, proposes that the injection can be done in the overlapping areas of angle L and angle M. It seeks to decrease the adverse reactions of BTX‐A treatment for MMP. However, even with an accurate injection site, the uncertain level of drug diffusion can still produce aftereffects. In clinical practice, clinicians typically use direct injection or retrograde injection to inject the masseter based on their own experience or habits [33]. This is likely to result in unequal drug diffusion. Some academics once proposed that the asymmetric smile is one of the common untoward reactions caused by the injection of BTX‐A into the masseter due to the diffusion of medication to the risorius muscle [34]. The risorius typically passes through the part anterior to the one‐third of the superficial masseter with an unfixed course direction [35]. It is likely to be located under the overlapping area of angle M of the safe triangular zone, while point M is close to the course region. Therefore, injections in the overlapping area of angle M should adopt direct injection to make the drug directly act on the middle masseter. At the same time, the deep inferior tendon (DIT) should be used to prevent the drug from diffusing toward the risorius, facial nerve, and other structures. The areas around angle L and angle F of the safe triangular zone have fewer courses of the critical structure, and retrograde injection can be done here. Thus, the drug can evenly diffuse to the superficial masseter and deep masseter to ensure that the drug fully acts on all layers of the masseter and reduces the occurrence of PMB.

It is also crucial to address the potential effects of BTX injections on bone tissue. Recent studies have highlighted that repeated BTX injections can induce adverse changes in mandibular bone quality, such as bony changes in the condyle and angle, primarily due to muscle atrophy and reduced mechanical loading [7, 8, 35]. While our proposed safe zone is aimed at minimizing injury to soft tissue structures (nerves and vessels), it does not negate the physiological impact of muscle unloading on the underlying bone. Therefore, clinicians should be aware that even with a precise “safe zone” injection, the risk of long‐term bony changes persists, and patients should be counseled accordingly.

Finally, this study has several limitations. First, the use of embalmed cadavers may not perfectly reflect the tissue texture and volume of living subjects due to potential tissue shrinkage during fixation. Second, the study included specimens with a wide age range (from 35 to 87 years). Aging is known to cause muscle atrophy and a reduction in cross‐sectional area [36, 37]. Although skeletal landmarks remain relatively constant, severe muscle atrophy in elderly specimens might slightly influence the localized measurements of the safe region compared to a younger patient population.

To sum up, this experiment suggests a three‐point injection strategy for the masseter based on anatomy and by combining the heatmap with the masseteric structure: retrograde injection can be done in the area of angle L of the safe triangular zone of △LMF; direct injection can be done in the area of angle M; and retrograde injection or oblique retrograde injection should be done in the most prominent part of the masseter from a point selected within angle F of the safe zone. This approach is expected to improve injection accuracy and reduce the occurrence of complications.

## 5. Conclusions

The safe triangular zone and three‐point injection approach proposed in this study by deep anatomical research are conducive to reducing the risk of harm to key tissues during the microplastic injection of the masseter. Compared to traditional injection methods, this study establishes a coordinate system with equal proportions while combining it with landmarks to localize. It employs a heatmap to represent the occurrence of the various structures of the superficial masseter to identify injection sites. It increases injection accuracy and allows clinicians to make individualized adjustments to treatment regimens based on patients’ facial characteristics and treatment responses. Furthermore, this strategy will considerably reduce the occurrence of complications while providing a firm anatomical foundation and clinical guidance for the minimally invasive treatment of MMP. Nonetheless, due to the formaldehyde‐fixed gross specimens utilized in this study and the small sample size, it does not distinguish between genders. Future research can involve a broader range of patient groups to explore the impact of different injection methods on the treatment effect of subjects of different genders.

## Funding

This study was funded by the 2023 Provincial College Student Innovation and Entrepreneurship Training Program of Chengdu Medical College (S202413705106), the 2025 Sichuan Provincial College Students’ Innovation and Entrepreneurship Training Program (S202513705091), and the Technical Service Research Project of Chengdu Medical College (CMC He [2023] No. 881)

## Ethics Statement

This study was approved by the Ethics Committee of Chengdu Medical College (2023NO.85).

## Conflicts of Interest

The authors declare no conflicts of interest.

## Data Availability

Due to ethical restrictions related to human anatomical research, the raw data supporting the conclusions of this study are not publicly available. However, deidentified data may be obtained from the corresponding author upon reasonable request and with approval from the Biomedical Ethics Committee of Chengdu Medical College.
